# Genetic Variants on Chromosome 1p13.3 Are Associated with Non-ST Elevation Myocardial Infarction and the Expression of *DRAM2* in the Finnish Population

**DOI:** 10.1371/journal.pone.0140576

**Published:** 2015-10-28

**Authors:** Perttu P. Salo, Satu Vaara, Johannes Kettunen, Matti Pirinen, Antti-Pekka Sarin, Heikki Huikuri, Pekka J. Karhunen, Markku Eskola, Kjell Nikus, Marja-Liisa Lokki, Samuli Ripatti, Aki S. Havulinna, Veikko Salomaa, Aarno Palotie, Markku S. Nieminen, Juha Sinisalo, Markus Perola

**Affiliations:** 1 Genomics and Biomarkers Unit, Department of Health, National Institute for Health and Welfare, Helsinki, Finland; 2 Institute for Molecular Medicine Finland (FIMM), University of Helsinki, Helsinki, Finland; 3 Children's Hospital, Helsinki University Central Hospital, University of Helsinki, Helsinki, Finland; 4 Division of Cardiology, Heart and Lung Center HUS, Helsinki University Central Hospital, Helsinki, Finland; 5 Institute of Clinical Medicine, Department of Internal Medicine, University Central Hospital and University of Oulu, Oulu, Finland; 6 School of Medicine, University of Tampere, Tampere, Finland; 7 Fimlab Laboratories Ltd, Tampere University Hospital Region, Tampere, Finland; 8 Heart Center, Tampere University Hospital and University of Tampere, School of Medicine, Tampere, Finland; 9 Transplantation Laboratory, Haartman Institute, University of Helsinki, Helsinki, Finland; 10 Health Monitoring Unit, Department of Health, National Institute for Health and Welfare, Helsinki, Finland; 11 Wellcome Trust Sanger Institute, Hinxton, Cambridge, United Kingdom; 12 Broad Institute, Cambridge, MA, United States of America; 13 The Estonian Genome Center of the University of Tartu, Tartu, Estonia; Universite de Montreal, CANADA

## Abstract

Myocardial infarction (MI) is divided into either ST elevation MI (STEMI) or non-ST elevation MI (NSTEMI), differing in a number of clinical characteristics. We sought to identify genetic variants conferring risk to NSTEMI or STEMI by conducting a genome-wide association study (GWAS) of MI stratified into NSTEMI and STEMI in a consecutive sample of 1,579 acute MI cases with 1,576 controls. Subsequently, we followed the results in an independent population-based sample of 562 cases and 566 controls, a partially independent prospective cohort (N = 16,627 with 163 incident NSTEMI cases), and examined the effect of disease-associated variants on gene expression in 513 healthy participants. Genetic variants on chromosome 1p13.3 near the damage-regulated autophagy modulator 2 gene *DRAM2* associated with NSTEMI (rs656843; odds ratio 1.57, P = 3.11 × 10^−10^) in the case-control analysis with a consistent but not statistically significant effect in the prospective cohort (rs656843; hazard ratio 1.13, P = 0.43). These variants were not associated with STEMI (rs656843; odds ratio, 1.11, P = 0.20; hazard ratio 0.97, P = 0.87), appearing to have a pronounced effect on NSTEMI risk. A majority of the variants at 1p13.3 associated with NSTEMI were also associated with the expression level of *DRAM2* in blood leukocytes of healthy controls (top-ranked variant rs325927, P = 1.50 × 10^−12^). The results suggest that genetic factors may in part influence whether coronary artery disease results in NSTEMI rather than STEMI.

## Introduction

Myocardial infarction (MI) is diagnosed as either ST elevation MI (STEMI) or non-ST elevation MI (NSTEMI) based on specific electrocardiogram (ECG) patterns [1]. In most cases STEMI results from complete occlusion of a coronary artery [2] whereas NSTEMI is caused by partial or transient blockage [3]. In either case, prolonged ischemia ultimately leads to cell death. Both differences and similarities in prognoses for NSTEMI and STEMI have been reported, depending on follow-up time and study sample. [4, 5] Despite improvements in treatment, ischemic heart disease remains the most common cause of death world-wide [6].

STEMI and NSTEMI differ in a number of clinically relevant characteristics. Compared to STEMI, NSTEMI tends to present with greater minimal luminal area in the culprit artery, smaller length of plaque rupture [7], and smaller infarct size [8]. The ruptured plaques are also found to be somewhat smaller and more heavily calcified in NSTEMI [9]. Interestingly, recurrent infarctions are often of the same type, suggesting that some individuals may be particularly susceptible to either STEMI or NSTEMI [10]. Although MI is moderately heritable [11] and a significant number of coronary artery disease (CAD) or MI risk loci have been identified [12], the possible differences in genetic risk factors between STEMI and NSTEMI have been virtually unexplored.

To identify genetic variants conferring susceptibility to STEMI or NSTEMI, we conducted a genome-wide association study (GWAS) of MI with stratification into NSTEMI and STEMI. We followed the results in a case-control sample and a prospective cohort. Finally, we examined the correlation of the disease-associated variants with gene expression levels in blood leukocytes in a sample of healthy individuals. All participants of this study were Finns.

## Results

### Discovery GWAS

**Fig 1:**
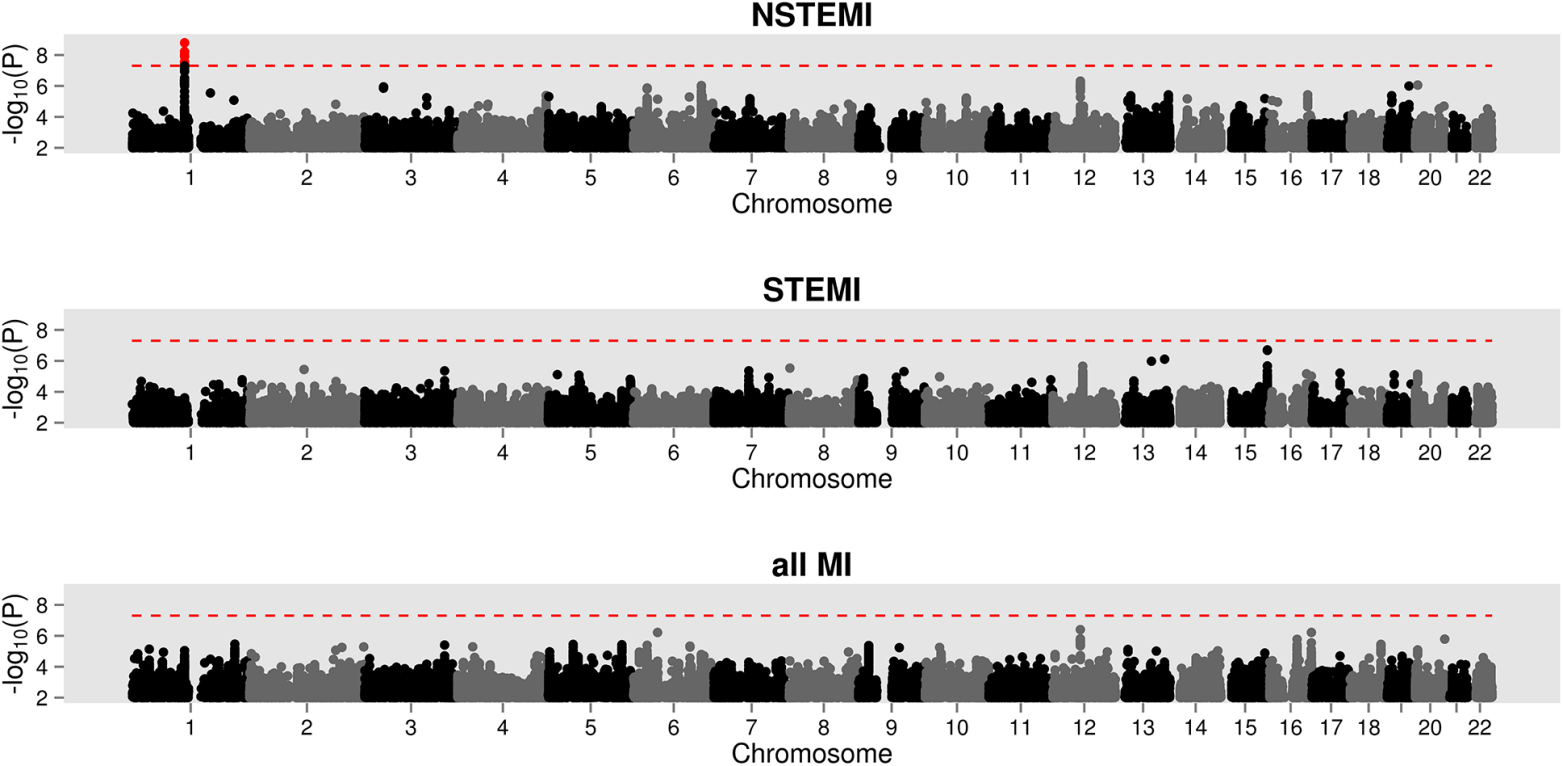
Summary of the genome-wide association results comparing healthy controls with only NSTEMI, only STEMI, or all acute MI cases. Variants reaching the genome-wide significance threshold P < 5 × 10^−8^, marked with the dashed line, are shown in red.

The characteristics of the discovery sample are presented in Table 1. Fig 1 and Table 2 summarize the GWAS P-values for MI, STEMI and NSTEMI. We detected a novel genome-wide significant association on chromosome 1p13.3 for NSTEMI with two directly genotyped and 14 imputed variants passing the pre-specified significance threshold of P < 5 × 10^−8^. The most statistically significant directly genotyped variant was rs656843 with P = 1.22 × 10^−8^ (odds ratio [OR] for the minor allele, 1.63 [95% CI 1.38 to 1.93]) and the most significantly associated imputed variant was rs2764553 (OR for the minor allele, 1.62 [95% CI 1.24 to 1.63]; P = 1.63 × 10^−9^). None of the SNPs reached genome-wide significance when testing for association with STEMI or all MI cases. We did not observe inflation in the test statistics and the genome-wide inflation factors for the directly genotyped SNPs were close to 1 for each of the phenotypes we tested (λ_MI_ = 1.03, λ_STEMI_ = 1.03, λ_NSTEMI_ = 1.02).

**Table 1:** Sample characteristics.

Sample	Stratum	N	Males	Age (years)	BMI (kg/m^2^)	Diabetes	Current smoking
Discovery	All MI	1,579^a^	1,102 (69.8)	65.6 ± 11.9	26.9 ± 6.6	345 (21.8)^1^	517 (32.7)^15^
	STEMI	614	451 (73.5)	63.0 ± 12.0	26.7 ± 6.7	97 (15.8)	252 (41.5)^7^
	NSTEMI	962	649 (67.5)	67.2 ± 11.6	27.0 ± 6.5	248 (25.8)^1^	264 (27.7)^8^
	Controls	1,576	915 (58.1)	57.5 ± 11.0	26.9 ± 5.3	117 (7.4)	382 (24.2)
Replication I	All MI	562	340 (60.5)	69.9 ± 11.6	na.	132 (23.5)^8^	102 (18.1)^11^
	STEMI	173	112 (64.7)	66.6 ± 12.3	na.	31 (17.9)^1^	41 (23.7)^3^
	NSTEMI	389	228 (58.6)	71.4 ± 11.0	na.	101 (30.0)^7^	61 (15.7)^8^
	Controls	566	275 (48.6)	49.5 ± 12.9	26.9 ± 4.5	41 (7.2)	106 (18.7)
Replication II	All MI	484^b^	348 (71.9)	65.81 ± 9.69	28.72 ± 4.3^5^	82 (16.94)	170 (35.12)
	STEMI	99	78 (78.79)	62.32 ± 10.25	29.27 ± 3.94^1^	17 (17.17)	39 (39.39)
	NSTEMI	163	111 (68.1)	67.05 ± 9.11	28.33 ± 4.2^1^	31 (19.02)	51 (31.29)
	Controls	16,143	8,904 (45.8)	58.46 ± 12.80	26.57 ± 4.69^45^	916 (5.67)	4,006 (24.82)

^**a**^ Includes 3 MI cases with unspecified ST-segment status

^**b**^ Includes 222 MI cases with unspecified ST-segment status

Mean ± standard deviation is shown for continuous variables and number (%) for categorical variables. The number of patients with missing information is shown in upper index.

BMI, body mass index;

MI, myocardial infarction;

STEMI, ST elevation myocardial infarction;

NSTEMI, non-ST elevation myocardial infarction;

na. not available

**Table 2:** Summary of the genome-wide significant association results on chromosome 1p13.3.

Variant	Position	Alleles ^a^	MAF ^Controls^	MAF ^NSTEMI^	OR ^NSTEMI^	P ^NSTEMI^	MAF ^STEMI^	OR ^STEMI^	P ^STEMI^	MAF ^All MI^	OR ^All MI^	P ^All MI^
rs685377^i^	111687613	T/G	0.20	0.26	1.53 (1.21–1.58)	4.32 × 10^−8^	0.21	1.12 (0.91–1.26)	0.20	0.24	1.31 (1.11–1.42)	5.50 × 10^−5^
rs613915^i^	111691009	A/C	0.20	0.26	1.57 (1.23–1.62)	9.29 × 10^−9^	0.20	1.09 (0.88–1.23)	0.32	0.23	1.31 (1.12–1.42)	4.45 × 10^−5^
rs586995	111692440	G/A	0.20	0.26	1.53 (1.31–1.79)	4.91 × 10^−8^	0.21	1.12 (0.95–1.33)	0.19	0.24	1.31 (1.15–1.49)	5.11 × 10^−5^
rs1093472^i^	111696753	G/T	0.20	0.26	1.54 (1.21–1.58)	4.25 × 10^−8^	0.21	1.12 (0.91–1.26)	0.20	0.24	1.31 (1.11–1.42)	5.46 × 10^−5^
rs591100^i^	111697305	G/A	0.20	0.26	1.54 (1.21–1.58)	4.24 × 10^−8^	0.21	1.12 (0.91–1.26)	0.20	0.24	1.31 (1.11–1.42)	5.46 × 10^−5^
rs669758^i^	111699202	A/C	0.20	0.26	1.54 (1.21–1.58)	4.21 × 10^−8^	0.21	1.12 (0.91–1.26)	0.20	0.24	1.31 (1.11–1.42)	5.44 × 10^−5^
rs680025^i^	111704797	C/G	0.20	0.26	1.54 (1.21–1.59)	4.11 × 10^−8^	0.21	1.12 (0.91–1.26)	0.20	0.24	1.31 (1.11–1.42)	5.39 × 10^−5^
rs679407^i^	111704923	G/C	0.20	0.26	1.54 (1.21–1.59)	4.11 × 10^−8^	0.21	1.12 (0.91–1.26)	0.20	0.24	1.31 (1.11–1.42)	5.39 × 10^−5^
rs11440587^i^	111707225	A/AT	0.20	0.26	1.54 (1.21–1.58)	4.05 × 10^−8^	0.21	1.12 (0.91–1.26)	0.20	0.24	1.31 (1.12–1.42)	5.16 × 10^−5^
rs629006^i^	111714400	G/A	0.20	0.26	1.54 (1.21–1.59)	3.88 × 10^−8^	0.21	1.12 (0.91–1.26)	0.20	0.24	1.31 (1.12–1.42)	5.28 × 10^−5^
rs675874^i^	111720775	G/T	0.20	0.26	1.54 (1.22–1.59)	3.02 × 10^−8^	0.21	1.12 (0.91–1.26)	0.21	0.24	1.31 (1.12–1.42)	4.98 × 10^−5^
rs641379^i^	111728285	T/C	0.20	0.26	1.54 (1.22–1.59)	2.71 × 10^−8^	0.21	1.12 (0.91–1.25)	0.21	0.24	1.31 (1.12–1.42)	4.83 × 10^−5^
rs2764553^i^	111732303	C/A	0.19	0.25	1.62 (1.24–1.63)	1.63 × 10^−9^	0.20	1.11 (0.89–1.24)	0.27	0.23	1.33 (1.12–1.43)	2.11 × 10^−5^
rs2484459^i^	111734126	G/C	0.15	0.21	1.66 (1.26–1.69)	5.99 × 10^−9^	0.16	1.07 (0.86–1.24)	0.48	0.19	1.33 (1.13–1.47)	9.48 × 10^−5^
rs656843	111736672	G/A	0.15	0.21	1.63 (1.38–1.93)	1.22 × 10^−8^	0.16	1.07 (0.88–1.29)	0.50	0.19	1.33 (1.15–1.54)	1.27 × 10^−4^
rs142769559^i^	111737998	G/GAAGC	0.16	0.22	1.59 (1.24–1.65)	3.90 × 10^−8^	0.17	1.12 (0.9–1.27)	0.25	0.20	1.33 (1.13–1.46)	6.77 × 10^−5^

^a^ Major/minor

^i^ Imputed variant

MAF, minor allele frequency;

OR, odds ratio;

MI, myocardial infarction;

STEMI, ST elevation myocardial infarction;

NSTEMI, non-ST elevation myocardial infarction

Statistical significance tested using logistic regression setting age, sex, and the first ten genomic principal components as covariates. The 95% confidence interval is reported in parentheses for the OR estimates.

**Fig 2:**
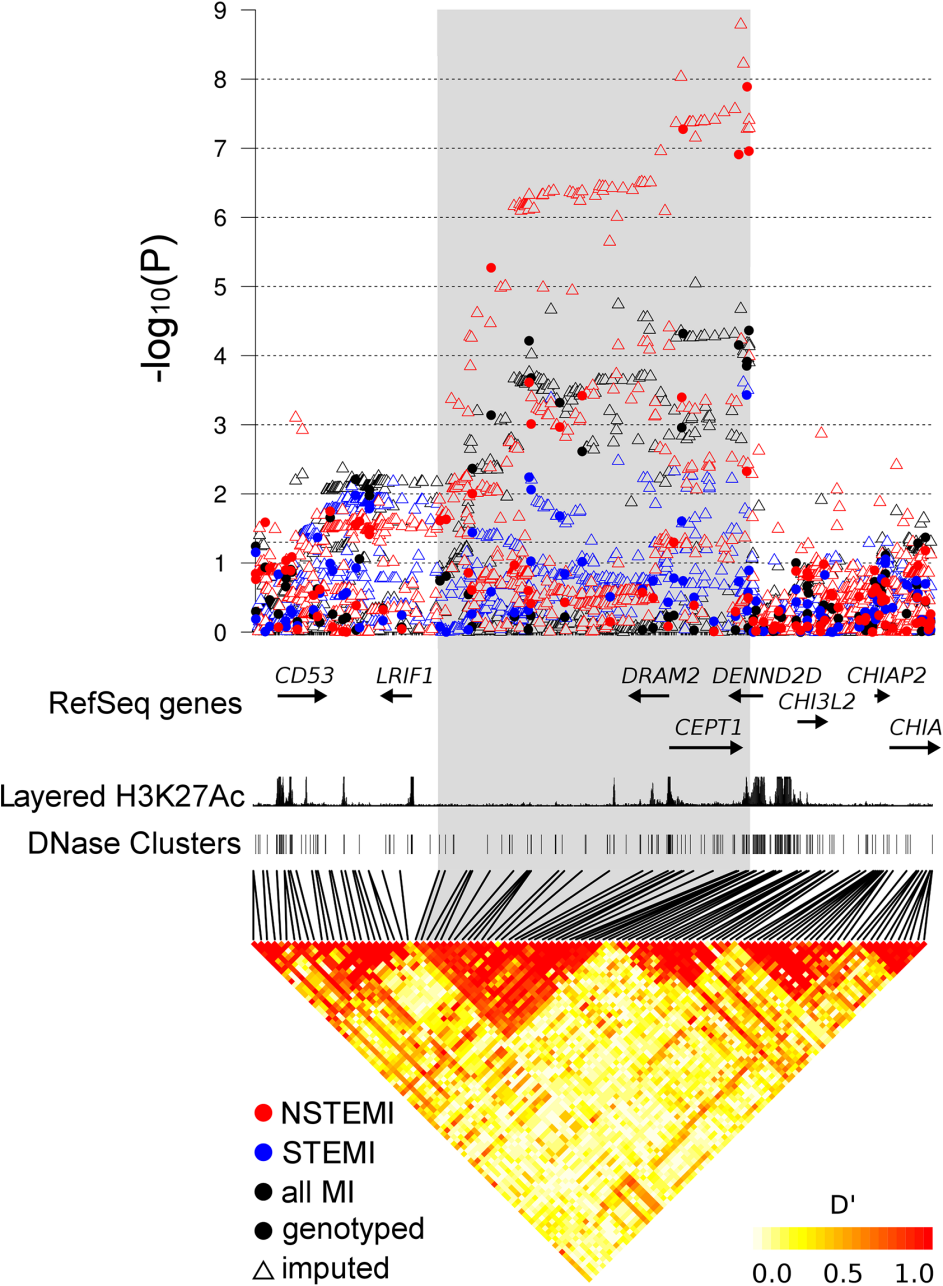
Association of genetic variants with the MI phenotypes and the LD structure at 1p13.3 (GRCh37 Chromosome 1:111,397,480–111,863,701). The P-values for both imputed and genotyped variants are depicted, while the LD is calculated from the genotyped SNPs only. Known genes from the RefSeq database are shown together with potential regulatory regions marked by histone 3 lysine 27 acetylation (Layered H3K27Ac) and DNase I hypersensitivity clusters from the Encode consortium.

The variants at 1p13.3 associated with NSTEMI are located within an LD block approximately 240 kbp in size, containing the genes *DRAM2*, *CEPT1* and the 3' end of *DENND2D* (Fig 2). Rs1335645 between *CEPT1* and *DENND2D* has previously been reported to associate with plasma gammaglutamyltransferase (GGT) levels [13]. Furthermore, rs599839 1.9 Mbp upstream of rs656843 near *PSRC1* and *SORT1* has been reported to be associated with both blood low-density lipoprotein cholesterol (LDL-C) levels and risk for CAD [14]. We thus tested rs656843 for association with GGT and LDL-C in 1,575 samples used as controls in the discovery sample but found no association (PGGT = 0.11; PLDL-C = 0.22; data not shown). Additionally, we calculated the correlation between rs656843 and these SNPs from the imputed data to confirm its independence of these two previously reported SNPs (r2 < 0.05).

### Replication and meta-analysis

We selected the most statistically significant directly genotyped SNP rs656843 for replication. Characteristics of the replication samples are presented in Table 1 and the results of the association tests in Table 3. We combined the results from the case-control samples in both fixed- and random-effects meta-analysis using directly genotyped data. We did not observe significant heterogeneity (*I* ^*2*^ < 0.10) and report the values obtained from the fixed-effects meta-analysis.

**Table 3:** Association P-values and effect-size estimates for rs656843 in the discovery and replication samples.

Sample	MAF ^Unaffected^	MAF ^NSTEMI^	OR ^NSTEMI^	P ^NSTEMI^	MAF ^STEMI^	OR ^STEMI^	P ^STEMI^	MAF ^All MI^	OR ^All MI^	P ^All MI^
Discovery^a^	0.15 (1,576)	0.21 (962)	1.63 (1.38–1.93)	1.22 × 10^−8^	0.16 (614)	1.07 (0.88–1.29)	0.50	0.19 (1,579)	1.33 (1.15–1.54)	1.27 × 10^−4^
Replication I^b^	0.14 (566)	0.19 (389)	1.44 (1.12–1.86)	0.0049	0.17 (173)	1.27 (0.91–1.77)	0.16	0.18 (562)	1.38 (1.10–1.74)	0.0062
Case-control combined^c^	0.15 (2,142)	0.20 (1,351)	1.57 (1.36–1.81)	3.11 × 10^−10^	0.16 (787)	1.11 (0.94–1.31)	0.20	0.19 (2,141)	1.34 (1.19–1.52)	2.58 × 10^−6^
Replication II^d^	0.17 (16,143)	0.18 (163)	1.13^e^ (0.84–1.51)	0.43	0.16 (99)	0.97^e^ (0.65–1.43)	0.87	0.16 (484)	1.00^e^ (0.84–1.19)	0.98

^a,b^ Case-control sample

^c^ Discovery and replication sample I combined using meta-analysis

^d^ Prospective sample

^e^ Hazard ratio

MAF, minor allele frequency;

OR, odds ratio;

MI, myocardial infarction;

STEMI, ST elevation myocardial infarction;

NSTEMI, non-ST elevation myocardial infarction;

na., not applicable

Case-control samples analyzed using logistic regression including age, sex, and the first ten genomic principal components (the discovery sample) or no covariates (Replication sample I) in the model. Replication sample II analyzed using the Cox proportional hazards model stratified by study year, geographical region, and genotyping batch with gender, systolic blood pressure, blood pressure medication, total cholesterol, HDL-cholesterol, smoking and diabetes used as covariates. Sample sizes (for the MAFs) and 95% confidence intervals (for the OR/hazard ratio estimates) are reported in parentheses.

First we tested rs656843 for association with the MI phenotypes in the replication sample I. Similar to the discovery sample, rs656843 was associated with NSTEMI (389 cases, 566 controls; OR for the minor allele, 1.44 [95% CI 1.12 to 1.86]; P = 0.0049) but not with STEMI (173 cases, 566 controls; OR 1.27 [95% CI 0.91–1.77]; P = 0.16). When comparing all MI cases with controls in the replication sample I the association of rs656843 was also statistically significant (562 cases, 566 controls; OR 1.38 [95% CI 1.10 to 1.74]; P = 0.0062).

Next we combined the results of the GWAS and the replication sample I to get a summary of the association of rs656843 with MI across the case-control data. The overall most significant association was with NSTEMI when we combined both discovery and replication samples using meta-analysis (OR 1.57 [95% CI 1.36–1.81]; P = 3.11 × 10^−10^). Rs656843 was not associated with STEMI in the meta-analysis of the discovery and replication samples (OR 1.11 [95% CI 0.94–1.31]; P = 0.20). Accordingly, the association of rs656843 with unspecified MI did not reach genome-wide significance when the two case-control samples were analyzed together (OR 1.34 [95% CI 1.19–1.52]; P = 2.58 × 10^−6^).

To complement the results from the case-control cohorts, we tested rs656843 for association with MI, NSTEMI, and STEMI in the prospective replication sample II. The association of rs656843 with NSTEMI, STEMI or MI was not statistically significant, although the estimate of the hazard ratio for NSTEMI (HR 1.13 [95% CI 0.84–1.51]) was consistent with the two case-control samples in direction of association. To assess the statistical power of the prospective sample, we tested 50 published MI/CAD risk SNPs present in our data for association with incident MI or NSTEMI. Depending on the phenotype tested, either 9 (MI) or 2 (NSTEMI) of the SNPs replicated at P < 0.05 (data not shown, SNPs listed in Table A in S1 File).

As both the discovery and replication samples consisted of Finns, we next wanted to study the association of rs656843 with MI or relevant traits in non-Finnish populations. Two large publicly available GWAS meta-analyses by the CARDIoGRAM [15] and C4D [16] consortia had tested the association of rs656843 with CAD in multiple populations. The CARDIoGRAM meta-analysis covered 16,693 cases and 55,947 controls from 22 European cohorts. In C4D, the meta-analysis combined results from two European and two South-Asian cohorts containing altogether 15,379 cases and 15,026 controls. Rs656843 was not associated with risk for CAD in either of the two meta-analyses.

The association of rs656843 was statistically significant for NSTEMI and not for STEMI in the discovery and replication I samples, suggesting the risk conferred by rs656843 may not be equal for these two alternative subtypes of MI. To formally address this difference in association, we calculated posterior probabilities for statistical models where rs656843 provided either no risk whatsoever, the same risk for NSTEMI and STEMI, a related but attenuated risk for STEMI, or risk only for NSTEMI. Assuming a uniform prior, the model with the effect specific to NSTEMI had the highest posterior probability (0.71) followed by the model with a related but attenuated effect for STEMI (posterior probability = 0.29). The null model with no effect (posterior probability = 3.10 × 10^−8^) and the model with the same effect for both NSTEMI and STEMI (posterior probability = 0.0013) were the least favored by the data.

### Association with gene expression

The variants associated (P < 0.05) with NSTEMI at the 1p13.3 locus are not predicted by the Ensembl VEP to change the structure of a protein (Ensembl Variant Effect Predictor, database version 81.37).[17] The causal variant(s) may therefore act by altering gene expression, change the nucleotide sequence of a noncoding RNA molecule or not be among the variants tested for association. As we had used a dense imputation reference and the region does not harbor conserved noncoding RNA genes, we considered the first option to be likely.

**Fig 3:**
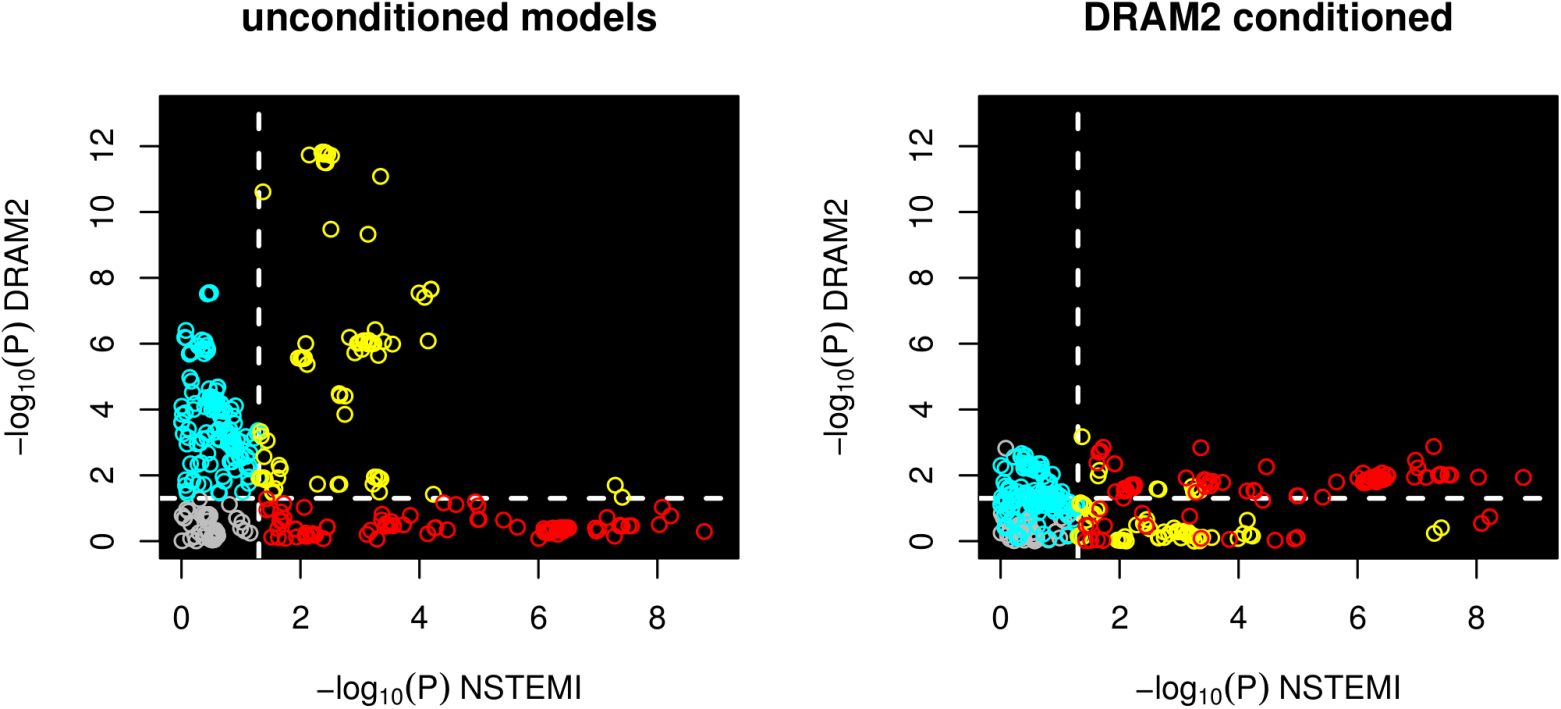
Association of genetic variants at 1p13.3 with *DRAM2* expression and NSTEMI. P-values for association with *DRAM2* expression are presented both for the basic model (left-hand side) and the model conditioned for rs325927 (right-hand side). The white dashed lines are drawn at P = 0.05 and divide the areas into quadrants. The variants are colored based on the quadrant they fall into under the unconditioned analysis model.

To identify *cis* regulatory effects, we tested the variants at the 1p13.3 locus for association with *DRAM2* and *CETP1* expression levels (no expression probes were available for *DENND2D*). The results of these tests are summarized in Table 4 and Fig 3. We detected a strong association with *DRAM2* expression level in blood leukocytes (expression probe ILMN_1808634; the most statistically significant variant rs325927, P = 1.50 × 10^−12^) and, after conditioning on the top SNP, a weaker secondary association signal (rs11102218, P = 6.70 × 10^−4^). The association of SNPs at 1p13.3 with NSTEMI overlaps both the primary and secondary association signals for *DRAM2* expression. However, rs656843 was not associated with *DRAM2* expression in either of the models. Furthermore, for the primary signal, NSTEMI risk-increasing alleles were associated with higher *DRAM2* expression, while the opposite was true for the SNPs constituting the secondary signal. Finally, we tested rs656843 genome-wide for association with all of the probes contained on the expression array in order to identify possible *trans* effects. None of the tests were statistically significant when corrected for the number of probes in either chromosome 1 alone or for all probes genome-wide.

**Table 4:** Association of SNPs with *DRAM2* expression and NSTEMI. Results are shown for the most statistically significant imputed and directly genotyped variants for each of the three phenotypes tested (*DRAM2* expression, *DRAM2* expression conditioned for rs325927, and NSTEMI).

Phenotype	Lead variant	Alleles ^a^	beta ^*DRAM2*^	P ^*DRAM*^	beta ^*DRAM2* conditioned^	P ^*DRAM2* conditioned^	OR ^NSTEMI^	P ^NSTEMI^
*DRAM2*	rs325927^i^	C/T	0.080 (0.059–0.102)	1.50 × 10^−12^	na.	na.	1.221 (1.065–1.4)	0.00421
*DRAM2*	rs657801	T/C	0.079 (0.057–0.100)	3.08 × 10^−12^	0.033 (-0.028–0.094)	0.291	1.223 (1.066–1.404)	0.00397
*DRAM2* conditioned	rs11102218^i^	G/A	0.078 (0.056–0.101)	2.43 × 10^−11^	0.047 (0.02–0.073)	6.70 × 10^−4^	0.874 (0.766–0.996)	0.0428
*DRAM2* conditioned	rs947633	A/G	-0.019 (-0.052–0.013)	0.237	-0.05 (-0.081–0.018)	0.00199	1.255 (1.033–1.526)	0.0223
NSTEMI	rs2764553^i^	A/C	0.009 (-0.018–0.036)	0.509	-0.036 (-0.064–0.008)	0.0116	1.622 (1.386–1.898)	1.63 × 10^−9^
NSTEMI	rs656843	C/T	0.025 (-0.004–0.054)	0.932	-0.016 (-0.046–0.014)	0.288	1.647 (1.39–1.951)	8.25 × 10^−9^

^a^ Effect/other

^i^ Imputed variant;

*DRAM2*, damage-regulated autophagy modulator 2;

NSTEMI, non-ST elevation myocardial infarction;

OR, odds ratio;

CI, confidence interval;

na., not applicable

Statistical significance tested using logistic regression including age, sex, and the first ten genomic principal components (NSTEMI) or no covariates (*DRAM2* expression) in the model. For consistency, all statistics reported for the imputed dataset.

Several of the variants associated with both NSTEMI and *DRAM2* expression in *cis* are predicted by the Ensembl VEP to have potential regulatory effects. Rs644081 is located at a splice junction of a non-canonical non-coding *DRAM2* transcript (ENST00000477769) and multiple SNPs reside within predicted regulatory regions. None of the variants affect 3' untranslated regions, suggesting the detected regulatory effects are probably not driven e.g. by miRNA binding. Strong linkage disequilibrium at the locus, however, makes it difficult to rank any of the potentially functional variants as the top candidate.

### Association of previously reported CAD/MI risk loci with NSTEMI and STEMI

Our GWAS data included 50 SNPs previously reported as associated with either unstratified MI, CAD or coronary heart disease (CHD) at P < 5 × 10^−8^ [18]. For 16 of these we replicated the association with MI at the nominal significance level of P < 0.05 (Table A in S1 File). These 16 SNPs included 5 near *CDKN2A*/*CDKN2B* on chromosome 9p21, the first and most widely replicated GWAS finding for MI [19]. At this locus we replicated the association with a nearly identical effect size estimate (OR for the minor allele of rs10757278, 1.28 [95% CI 1.15–1.43]; P = 1.25 × 10^−5^) as compared to the originally reported association with early-onset MI (OR for the rs10757278 minor allele, 1.28 [95% CI 1.22 to 1.35]). None of the 50 SNPs showed statistically significant differences in allele frequencies between STEMI and NSTEMI patients after adjusting for the number of tests (X^2^ test P > 0.001). 3 SNPs were nominally significant with the most statistically significant difference observed for rs514659 in the *ABO* gene determining the ABO blood group (frequency of the minor allele C in STEMI patients 48.06% and in NSTEMI patients 43.26%; X^2^ P = 0.0066), originally reported to be specifically associated with MI in presence of CAD [20].

## Discussion

We conducted a GWAS of acute MI, NSTEMI and STEMI followed by replication in both case-control and prospective settings. We identified a novel genome-wide significant association between SNPs on chromosome 1p13.3 and risk for NSTEMI. The association of the top directly genotyped SNP rs656843 with NSTEMI and unspecified MI replicated in the independent case-control sample. Similar to most already published GWAS CAD/MI loci, the effect of rs656843 was consistent in direction but did not reach statistical significance for MI or NSTEMI in the prospective sample. The contrast between the case-control and prospective analysis results may stem from the different case ascertainment methods and sample sizes. The case-control replication sample was collected by a single study center and contained 389 NSTEMI cases, whereas the 163 incident NSTEMI cases in the prospective replication sample were identified from hospital discharge registries, diagnosed during routine clinical work in multiple hospitals. The case-control sample thus benefitted from both a larger number of cases and potentially more uniform diagnostic criteria. The association of rs656843 with STEMI was not statistically significant in any of the samples we studied. The data from the three study samples thus suggest rs656843 is associated with NSTEMI but not with STEMI.

The difference in association of rs656843 with NSTEMI and STEMI could in principle be explained simply by the different sample sizes and a corresponding lack of statistical power to detect an association with STEMI, given that the samples contained more NSTEMI than STEMI cases. To address this, we compared the posterior probabilities of four candidate models using an approach that properly accounts for the different amounts of information available for STEMI and NSTEMI. The comparison suggests the data are best explained by a model where rs656843 increases risk for NSTEMI only. A model with a related but attenuated effect on STEMI risk also provides a good fit, while a model where the effect of rs656843 on NSTEMI and STEMI risk was set equal fits the data poorly. The observed data thus support the interpretation that the effect of the 1p13.3 locus on MI risk is not the same for NSTEMI and STEMI and that the difference in association is not caused solely by different sample sizes.

Rs656843 was not associated with risk for CAD in two large international GWAS meta-analyses, one conducted in European and the other in South-Asian and European cohorts. Although these meta-analyses of CAD are not a direct replication of an association detected using NSTEMI as the endpoint, a considerable proportion of the CAD cases in both meta-analyses had been diagnosed with MI. Given the large sample sizes, it seems unlikely that phenotypic differences alone would explain the lack of association between rs656843 and CAD in the meta-analyses. The association detected in the present study may therefore depend on a genetic or environmental factor sufficiently common in Finland but rare in other European populations.

Genetic variants associated with NSTEMI on chromosome 1p13.3 are located within a 240 kbp long LD block. The region contains three genes, *DRAM2*, *CETP1* and *DENND2D*, all of which could potentially be causal. Variants associated with NSTEMI are associated with the expression of *DRAM2* and not with *CEPT1*, pointing towards *DRAM2* as causal. It must, however, be noted that the association of SNPs with NSTEMI is not exactly mirrored by their association with *DRAM2* expression. As expression quantitative trait loci are known to be ubiquitous across the genome [21], it is possible that the overlap between the NSTEMI and *DRAM2* associations is coincidental rather than causal.

The exact function of *DRAM2* remains to be elucidated, but it has been shown to be highly expressed in heart and to play a role in autophagy, the main cellular mechanism for recycling and degradation of organelles and long-lived proteins [22]. Silencing *DRAM2* inhibits autophagy under starvation [23] and attenuates p53-mediated cell death [24], although *DRAM2* is not directly regulated by p53 [25]. Autophagy is particularly important for postmitotic cells such as cardiomyocytes and induced in response to both intra- and extracellular stress conditions [26]. Depending on the context, it can either promote cell survival or contribute to cell death. Autophagy has also been suggested to affect myocardial sensitivity to ischemia-reperfusion injury [27], potentially connecting *DRAM2* function and a mechanism of cardiac damage. Further studies in e.g. myocardium are, however, required to show whether *DRAM2* can influence cardiac function.

We did not observe statistically significant differences between STEMI and NSTEMI patients for SNPs previously reported to be associated with MI, CAD or CHD at genome-wide significant level (Table A in S1 File). As most of the previously reported SNPs have been identified as associated with CAD and not specifically with MI, this implies that genetic variants affecting mechanisms relevant prior to plaque rupture, principally the development of CAD, confer similar risk for both NSTEMI and STEMI. Genetic differences between the two could mainly have to do with the acute response to plaque rupture and coronary artery occlusion. Interestingly, the most statistically significant difference between STEMI and NSTEMI patients for a previously reported SNP was at the *ABO* gene, originally identified as associated with risk for MI in presence of CAD [20]. This is particularly intriguing because the *ABO* locus is also the only genome-wide significant finding associated specifically with risk for MI independently of CAD.

Several limitations of the current study must be noted. First, as all of the study participants were Finns, the findings may not be directly applicable to other ethnic groups. Second, the sample sizes were moderately small, implying that the effect-size estimates of the novel findings may be inflated. This view is supported by the lack of statistically significant association of rs656843 with NSTEMI in the prospective setting. Although limited by the modest number of incident cases studied, the prospective setting gives a valuable realistic indication of the limited predictive value of rs656843 for incident MI or NSTEMI. Third, the controls in the replication sample I were on average approximately 20 years younger than the cases, resulting in loss of statistical power as some of the controls will likely suffer from MI in the future. Fourth, we used easily accessible blood leukocytes to study the correlation between DNA sequence variants and gene expression instead of potentially more relevant cell types such as cardiomyocytes or arterial endothelial cells.

The association of specific genetic variants preferentially with NSTEMI implies that whether an individual will get one form of acute MI rather than the other may partly be genetically influenced. This suggests future studies on the genetic basis of myocardial infarction could benefit from making the distinction between STEMI and NSTEMI. These two MI subtypes appear to share a majority of their genetic risk factors, but the differences may offer unique insights into the processes leading from coronary artery disease to myocardial injury.

## Materials and Methods

### Study cohorts

The MI cases of the discovery sample comprise all successfully genotyped Corogene study participants suffering from a spontaneous myocardial infarction related to ischaemia due to a primary coronary event (type 1 MI):[1, 28] either STEMI (N = 614), NSTEMI (N = 962), or MI with usnpecified ST-segment status (N = 3). Participants of the Corogene study were recruited from all consecutive patients assigned for coronary angiography at the Helsinki University Central Hospital between July 2006 and March 2008. 91.2% of the patients gave informed consent and were included in the Corogene study, described in detail elsewhere.[29] We defined ACS as an episode of typical acute chest pain and at least 50% stenosis in one or more coronary arteries. We further classified the patients as having UAP, NSTEMI or STEMI according to ischaemic ECG changes and elevated levels of cardiac biomarkers. We defined STEMI as acute chest pain with persistent ST-elevations and elevated cardiac biomarkers; NSTEMI as acute chest pain, ST-depression or T-inversions combined with elevated cardiac biomarkers; and UAP as acute chest pain, ST-depression or T-inversions combined with negative cardiac biomarkers.[30] We considered the patients having typical symptoms and ST-segment elevation, but no significant coronary obstruction as ST-elevation myocardial infarction patients, if they had large cardiac enzyme release (creatine kinase-MBmass >50 mg/l). From the ACS patients, only MI patients were included in this study and those suffering from UAP were excluded.

Patients in the replication sample I were participants of the Tampere Acute Coronary Syndrome Study (TACOS) conducted in the city of Tampere and 11 neighbouring municipalities, a population of 340,000 where practically all patients with ACS are admitted to Tampere University Hospital [31]. During January 2002 to March 2003, patients admitted to the emergency department of Tampere University Hospital presenting symptoms of ACS verified by elevated blood troponin I (cTnI>0.2 mg/L) value were recruited to the study. In addition, from September 2002 to March 2003, consecutive troponin I-negative patients with symptoms typical of UAP were recruited. Patients transferred from another department within the hospital or initially treated for ACS in other hospitals were excluded. The final study sample contained 998 MI patients (343 with STEMI, 655 with NSTEMI). Of these, 564 STEMI or NSTEMI patients had both sufficient phenotypic information and blood samples available and were selected for this study. The diagnostic criteria for the TACOS study sample were similar to those of the Corogene study and have been presented in detail in an earlier publication.[31]

Controls in the discovery sample, replication sample I and subjects in the gene expression tests were participants of the FINRISK 1992, 1997, 2002 and 2007 cohorts. FINRISK surveys have been conducted every 5 years to monitor the risk factors of chronic diseases [32, 33]. For each survey, a representative random sample was selected from 25–74 year old inhabitants in different regions in Finland, stratified so that persons were selected from both genders per each 10-year age group in separate regions. A total of 23,036 individuals participated in FINRISK 1992, 1997, 2002 or 2007, and gave written informed consent. For the discovery sample, we selected as controls FINRISK 1997, 2002 and 2007 study participants from the Helsinki-Vantaa region using risk set sampling [34]. For each case independently, we sampled two controls (if possible) from all participants who were of the same sex, of the same birth cohort (±5 years) and free of cardiovascular disease at least until the time at which the respective case was diagnosed with ACS. The sampling was done with replacement, so that it was possible for a participant to be selected as a control multiple times. For the replication sample I, we selected 613 healthy controls, of which 567 were successfully genotyped, from the FINRISK 1992, 1997, 2002 and 2007 participants born in Pirkanmaa, the same geographical region where the cases were recruited. As FINRISK sampling does not cover Pirkanmaa, each control has moved from Pirkanmaa to one of the FINRISK sampling regions.

A second replication sample was formed by combining all FINRISK participants with genotype data available, who were genotyped genome-wide for previous studies and imputed with the same methodology as used for the discovery sample. We defined the disease endpoints from hospital discharge registries and excluded the controls of the discovery GWAS sample and replication sample I, individuals with insufficient phenotype data, and participants suffering from prevalent MI. The final sample contained 16,627 participants including 163 cases of incident NSTEMI, 99 cases of incident STEMI and 222 cases of incident MI with unspecified ST-segment status; the median follow-up was 9.93 years.

Gene expression levels were measured from 513 participants recruited during 2007 to the Dietary, Lifestyle, and Genetic determinants of Obesity and Metabolic syndrome (DILGOM) study, an extension of the FINRISK 2007 study. 372 of these samples were also used as controls for the Corogene MI cases in the GWAS. Participants were asked to fast overnight (at least 10 hours) before donating blood samples.

### Genetic measurements

The genotypes for the GWAS were from an initial dataset consisting of 2,234 individuals from the Corogene study, 1,579 individuals selected as controls for the Corogene study sample and an additional 141 DILGOM participants. The samples were genotyped using the Illumina HumanHap 610-Quad SNP Array at the Wellcome Trust Sanger Institute, Cambridge, United Kingdom. We set missing genotypes with clustering probability < 95% and excluded SNPs with a genotyping success rate < 95%, minor allele frequency (MAF) < 1%, or P < 10^−6^ for an exact test of Hardy-Weinberg equilibrium. We also excluded individuals with a genotyping success rate < 95% and individuals with different reported and genotype-determined gender. We then estimated the pair-wise identity-by-descent for all pairs in the sample and excluded one from each pair of closely-related individuals. Finally, we retained only autosomal SNPs.

To augment the dataset with imputed variants, we pre-phased it with shapeIT v1 [35] and used IMPUTE v2.2.2 [36] with the 1000 Genomes Project integrated variant set (release v3, March 2012) for genotype imputation. Variants with imputation score < 0.6 or MAF < 5% were excluded. For the analysis of the case-control sample using only directly genotyped SNPs, we retained only the MI cases and controls and subjected the remaining sample to the same filters as previously with the MAF cutoff set at 5%. Additionally, we removed SNPs with P < 0.05 for Pearson's X2 test for genotype missingness between cases and controls. A total of 1,579 MI cases (962 NSTEMI, 614 STEMI, 3 MI with unspecified ST-segment status), 1,576 controls, 485,919 genotyped SNPs and 5,968,900 imputed variants passed quality control.

The replication sample I was genotyped using the Sequenom MassARRAY iPLEX platform at the Institute For Molecular Medicine Finland FIMM, Helsinki, Finland, following standard procedures. Genotype quality was assessed by genotyping 74 samples in duplicate with 100% concordance for both SNPs. A total of 1,131 participants (390 NSTEMI, 174 STEMI, 567 controls) were successfully genotyped. To form the replication sample II, we combined the genotypes of the FINRISK participants of the discovery sample with individuals genotyped on a variety of different genome-wide genotyping arrays for previous studies (Table B in S1 File), imputed using the same methods as were used for the discovery GWAS. This resulted in both genotypic and phenotypic information being available for 16,627 FINRISK participants not used as controls in the discovery and replication I samples.

Gene expression was measured from blood leukocytes using the Illumina HT-12 expression array as described previously [37]. Briefly, each sample was measured in duplicates and the probe signal intensities were background corrected and normalized so that the signal intensity distributions for all samples on all arrays were the same. Correlation between the two technical replicates was measured and 9 samples were excluded due to poor correlation. Finally, we log2-transformed the corrected and normalized probe intensities.

### Statistical methods

We calculated the genome-wide principal components for the discovery sample with EIGENSTRAT v4.2 [38] from 87,046 directly genotyped SNPs in approximate linkage equilibrium. For all association tests we used an additive genetic model. We analyzed the association of genetic variants with the MI phenotypes with logistic regression using PLINK v1.07 [39] for the directly genotyped SNPs and SNPTEST v2.4.0 [40] for the imputed variants. We compared either all MI patients or only STEMI or NSTEMI patients with the same set of controls using age, gender and the 10 first genomic principal components as covariates. For the replication sample I with controls matched for geographical area but not for age or gender, we did not use age or gender as covariates as recommended [41]. We combined the results of the logistic regression models with GWAMA v2.1 [42] using inverse-variance weighted fixed- and random-effects meta-analysis models. To compare association statistics between different MI types, we used a Bayesian model comparison analysis method described in detail elsewhere [43]. For the prospective replication sample II, we used the Cox proportional hazards model implemented in the "survival" package [44] for R v3.0.2 [45]. We stratified the analysis by study year, geographical region and genotyping batch setting gender, systolic blood pressure, blood pressure medication, total cholesterol, HDL-cholesterol, current smoking and prevalent diabetes as covariates. For analyzing the association of genetic variants with gene expression levels, we used SNPTEST v2.4.0 and linear regression with the probe intensities as the dependent variables.

### Ethics statement

This study was conducted according to the principles expressed in the Declaration of Helsinki and all participants gave their written informed consent. The study was approved by the Ethical Committee for Internal Medicine of the Hospital District of Helsinki and Uusimaa (Nr. 426/E5/05), the Ethical Issues Committee of KTL (Jan/23/1997, Nr. 38/96, and 87/2001), the Coordinating Ethics Committee of the Helsinki and Uusimaa Hospital District (229/E0/06), and the Ethics Committee at Tampere University Hospital (Nr. R02100).

## Supporting Information

S1 File Supplementary Tables A & B. (PDF)
