# Supplementary material for: Genetic Variants on Chromosome 1p13.3 Are Associated with Non-ST Elevation Myocardial Infarction and the Expression of DRAM2 in the Finnish Population
Source: PLoS One. 2015 Oct 28;10(10):e0140576. doi: 10.1371/journal.pone.0140576 (PMC4625034; doi:10.1371/journal.pone.0140576)
Supplement: S1 File — (PDF) [file pone.0140576.s001.pdf]

SUPPLEMENTAL MATERIAL.

Supplementary Table A. Association of previously reported loci with NSTEMI and STEMI.

SNP	Trait	Reported				Effect allele/Other allele	MI		STEMI		NSTEMI		Effect allele frequency		
		Gene(s)	Effect allele	OR (95% CI)	P[a]		OR (95% CI)	P	OR (95% CI)	P	OR (95% CI)	P	STEMI	NSTEMI	X^2 P[b]
rs10757278	MI	CDKN2A, CDKN2B	G	1.28 (1.22-1.35)	1.00 E-20	G/A	1.28 (1.15-1.43)	1.25 E-05	1.25 (1.09-1.44)	2.04E-03	1.3 (1.14-1.48)	1.13 E-04	0.45	0.45	9.86E-01
rs1333049	CHD	CDKN2A, CDKN2B	C	1.47 (1.27-1.70)	1.00 E-13	C/G	1.27 (1.14-1.43)	2.87 E-05	1.24 (1.08-1.44)	3.06E-03	1.29 (1.13-1.48)	2.17 E-04	0.45	0.45	9.56E-01
rs6922269	CHD	MTHFD1L	A	1.23 (1.15-1.33)	3.00 E-08	A/G	1.04 (0.91-1.18)	5.53 E-01	1.07 (0.9-1.26)	4.33E-01	1.03 (0.88-1.2)	7.38 E-01	0.24	0.23	6.21E-01
rs1333049	CHD	Intergenic	C	1.36 (1.27-1.46)	3.00 E-19	C/G	1.27 (1.14-1.43)	2.87 E-05	1.24 (1.08-1.44)	3.06E-03	1.29 (1.13-1.48)	2.17 E-04	0.45	0.45	9.56E-01
rs599839	CHD	PSRC1	A	1.29 (1.18-1.40)	4.00 E-09	A/G	1.04 (0.91-1.19)	5.25 E-01	0.98 (0.82-1.16)	7.89E-01	1.1 (0.94-1.28)	2.45 E-01	0.21	0.20	4.79E-01
rs501120	CHD	CXCL12	T	1.33 (1.20-1.48)	9.00 E-08	C/T	1.07 (0.93-1.24)	3.54 E-01	1.01 (0.83-1.22)	9.39E-01	1.11 (0.94-1.32)	2.19 E-01	0.16	0.17	2.83E-01
rs11206510	MI	PCSK9	T	1.15 (1.10-1.21)	1.00 E-08	C/T	0.94 (0.81-1.1)	4.42 E-01	1.03 (0.85-1.24)	7.99E-01	0.89 (0.75-1.07)	2.22 E-01	0.15	0.14	2.34E-01
rs646776	MI	CELSR2, PSRC1, SORT1	T	1.19 (1.13-1.26)	8.00 E-12	T/C	1.06 (0.93-1.2)	4.15 E-01	0.98 (0.83-1.15)	7.89E-01	1.12 (0.96-1.3)	1.59 E-01	0.21	0.20	3.34E-01
rs6725887	MI	WDR12	C	1.17 (1.11-1.23)	1.00 E-08	C/T	1.29 (1.09-1.53)	3.49 E-03	1.21 (0.97-1.51)	9.37E-02	1.28 (1.05-1.57)	1.43 E-02	0.12	0.13	5.24E-01
rs4977574	MI	CDKN2A, CDKN2B	G	1.29 (1.25-1.34)	3.00 E-44	G/A	1.27 (1.14-1.42)	1.39 E-05	1.23 (1.07-1.42)	2.95E-03	1.3 (1.14-1.48)	7.13 E-05	0.47	0.47	9.75E-01
rs1746048	MI	CXCL12	C	1.17 (1.11-1.24)	7.00 E-09	T/C	1.07 (0.93-1.24)	3.37 E-01	1.01 (0.84-1.22)	8.97E-01	1.11 (0.94-1.32)	2.09 E-01	0.16	0.17	3.10E-01
rs12526453	MI	PHACTR1	C	1.12 (1.08-1.17)	1.00 E-09	G/C	0.9 (0.8-1.01)	8.29 E-02	0.89 (0.76-1.04)	1.58E-01	0.9 (0.78-1.04)	1.60 E-01	0.27	0.27	9.66E-01
rs17465637	MI	MIA3	C	1.14 (1.10-1.19)	1.00 E-09	C/A	1.07 (0.94-1.21)	3.11 E-01	0.98 (0.84-1.15)	8.33E-01	1.11 (0.96-1.29)	1.51 E-01	0.26	0.24	1.85E-01

SNP	Trait	Reported				Effect allele/Other allele	MI		STEMI		NSTEMI		Effect allele frequency		
		Gene(s)	Effect allele	OR (95% CI)	P[a]		OR (95% CI)	P	OR (95% CI)	P	OR (95% CI)	P	STEMI	NSTEMI	X^2 P[b]
rs1122608	MI	LDLR	G	1.15 (1.10-1.20)	2.00 E-09	T/G	0.88 (0.77-1.01)	7.32 E-02	0.93 (0.78-1.11)	4.11E-01	0.83 (0.71-0.98)	2.49 E-02	0.20	0.19	3.98E-01
rs9982601	MI	SLC5A3, MRPS6, KCNE2	T	1.2 (1.14-1.27)	6.00 E-11	T/C	1.11 (0.95-1.3)	1.83 E-01	1.16 (0.95-1.42)	1.45E-01	1.09 (0.91-1.32)	3.46 E-01	0.15	0.15	8.36E-01
rs9818870	CHD	MRAS	T	1.15 (1.11-1.19)	7.00 E-13	T/C	1.03 (0.87-1.22)	7.22 E-01	1.14 (0.92-1.42)	2.37E-01	0.93 (0.76-1.14)	5.06 E-01	0.12	0.11	2.66E-01
rs3739998	CHD	KIAA1462	C	1.15 (1.11-1.20)	1.00 E-11	G/C	1.04 (0.94-1.16)	4.37 E-01	0.98 (0.85-1.13)	7.80E-01	1.09 (0.96-1.24)	1.87 E-01	0.43	0.46	1.13E-01
rs11748327	MI	Intergenic	?	1.25 (1.18-1.33)	5.00 E-13	T/C	0.94 (0.83-1.07)	3.77 E-01	0.98 (0.83-1.15)	7.67E-01	0.93 (0.8-1.08)	3.58 E-01	0.22	0.22	6.81E-01
rs1994016	CHD	ADAMTS7	C	1.19 (1.13-1.24)	5.00 E-13	T/C	0.88 (0.79-1)	4.29 E-02	0.89 (0.76-1.03)	1.18E-01	0.88 (0.76-1.01)	6.31 E-02	0.30	0.30	9.41E-01
rs514659	MI	ABO	C	1.21 (1.13-1.28)	8.00 E-09	C/A	1.11 (1-1.24)	5.76 E-02	1.25 (1.08-1.44)	2.07E-03	0.98 (0.86-1.12)	8.18 E-01	0.48	0.43	6.60E-03
rs646776	CHD	CELSR2, PSRC1, SORT1	T	1.14 (1.09-1.19)	6.00 E-10	T/C	1.06 (0.93-1.2)	4.15 E-01	0.98 (0.83-1.15)	7.89E-01	1.12 (0.96-1.3)	1.59 E-01	0.21	0.20	3.34E-01
rs1332844	CHD	PHACTR1	T	1.11 (1.07-1.15)	6.00 E-08	T/C	1.14 (1.02-1.28)	2.37 E-02	1.14 (0.98-1.32)	9.29E-02	1.16 (1.01-1.33)	3.19 E-02	0.31	0.31	8.89E-01
rs9349379	CHD	PHACTR1	?	NR	9.00 E-26	G/A	1.22 (1.1-1.36)	2.57 E-04	1.2 (1.04-1.38)	1.26E-02	1.23 (1.09-1.4)	1.26 E-03	0.47	0.48	8.88E-01
rs10953541	CHD	Intergenic	C	1.08 (1.05-1.11)	3.00 E-08	T/C	1.05 (0.93-1.19)	4.42 E-01	1.1 (0.94-1.29)	2.30E-01	1.01 (0.88-1.17)	8.64 E-01	0.27	0.25	1.46E-01
rs4977574	CHD	Intergenic	G	1.2 (1.16-1.25)	2.00 E-25	G/A	1.27 (1.14-1.42)	1.39 E-05	1.23 (1.07-1.42)	2.95E-03	1.3 (1.14-1.48)	7.13 E-05	0.47	0.47	9.75E-01
rs1412444	CHD	LIPA	T	1.09 (1.07-1.12)	3.00 E-13	T/C	1.12 (1.01-1.25)	3.74 E-02	1.09 (0.95-1.26)	2.20E-01	1.15 (1.01-1.31)	4.14 E-02	0.42	0.42	8.80E-01
rs2505083	CHD	KIAA1462	C	1.07 (1.04-1.09)	4.00 E-08	C/T	1.05 (0.94-1.17)	4.04 E-01	0.97 (0.85-1.12)	7.20E-01	1.09 (0.96-1.24)	1.89 E-01	0.44	0.47	9.97E-02
rs974819	CHD	PDGFD	T	1.07 (1.04-1.09)	2.00 E-09	C/T	0.95 (0.83-1.08)	4.19 E-01	1.03 (0.88-1.22)	6.87E-01	0.93 (0.8-1.08)	3.69 E-01	0.22	0.24	2.30E-01
rs4380028	CHD	ADAMTS7, MORF4L1	C	1.07 (1.05-1.10)	4.00 E-09	T/C	0.81 (0.71-0.91)	4.88 E-04	0.78 (0.67-0.91)	1.94E-03	0.83 (0.72-0.96)	1.17 E-02	0.25	0.27	4.08E-01

SNP	Trait	Reported				Effect allele/Other allele	MI		STEMI		NSTEMI		Effect allele frequency		
		Gene(s)	Effect allele	OR (95% CI)	P[a]		OR (95% CI)	P	OR (95% CI)	P	OR (95% CI)	P	STEMI	NSTEMI	X^2 P[b]
rs11206510	CHD	PCSK9	T	1.08 (1.05-1.11)	9.00 E-08	C/T	0.94 (0.81-1.1)	4.42 E-01	1.03 (0.85-1.24)	7.99E-01	0.89 (0.75-1.07)	2.22 E-01	0.15	0.14	2.34E-01
rs6725887	CHD	WDR12	C	1.14 (1.09-1.19)	1.00 E-09	C/T	1.29 (1.09-1.53)	3.49 E-03	1.21 (0.97-1.51)	9.37E-02	1.28 (1.05-1.57)	1.43 E-02	0.12	0.13	5.24E-01
rs2306374	CHD	MRAS	C	1.12 (1.07-1.16)	3.00 E-08	C/T	1.03 (0.87-1.22)	6.96 E-01	1.15 (0.92-1.43)	2.18E-01	0.93 (0.76-1.14)	4.99 E-01	0.12	0.11	2.65E-01
rs4977574	CHD	CDKN2A, CDKN2B	G	1.29 (1.23-1.36)	1.00 E-22	G/A	1.27 (1.14-1.42)	1.39 E-05	1.23 (1.07-1.42)	2.95E-03	1.3 (1.14-1.48)	7.13 E-05	0.47	0.47	9.75E-01
rs12413409	CHD	CYP17A1, CNNM2, NT5C2	G	1.12 (1.08-1.16)	1.00 E-09	A/G	1.05 (0.86-1.26)	6.48 E-01	0.92 (0.71-1.18)	5.03E-01	1.15 (0.92-1.44)	2.11 E-01	0.08	0.09	1.31E-01
rs1746048	CHD	CXCL12	C	1.09 (1.07-1.13)	3.00 E-10	T/C	1.07 (0.93-1.24)	3.37 E-01	1.01 (0.84-1.22)	8.97E-01	1.11 (0.94-1.32)	2.09 E-01	0.16	0.17	3.10E-01
rs964184	CHD	ZNF259, APOA5, APOA4, APOC3, APOA1	G	1.13 (1.10-1.16)	1.00 E-17	C/G	1 (0.86-1.16)	9.80 E-01	1.02 (0.84-1.24)	8.32E-01	0.99 (0.83-1.18)	8.86 E-01	0.15	0.15	9.06E-01
rs2895811	CHD	HHIPL1	C	1.07 (1.05-1.10)	1.00 E-10	C/T	1.1 (0.98-1.23)	9.17 E-02	1.08 (0.94-1.24)	3.04E-01	1.11 (0.98-1.26)	1.09 E-01	0.45	0.45	7.60E-01
rs12190287	CHD	TCF21	C	1.08 (1.06-1.10)	1.00 E-12	G/C	0.85 (0.76-0.96)	6.88 E-03	0.89 (0.77-1.03)	1.20E-01	0.84 (0.73-0.96)	1.11 E-02	0.40	0.39	5.90E-01
rs3825807	CHD	ADAMTS7	A	1.08 (1.06-1.10)	1.00 E-12	G/A	0.87 (0.77-0.97)	1.29 E-02	0.88 (0.76-1.02)	8.36E-02	0.85 (0.74-0.97)	1.73 E-02	0.33	0.33	8.66E-01
rs17465637	CHD	MIA3	C	1.14 (1.09-1.20)	1.00 E-08	C/A	1.07 (0.94-1.21)	3.11 E-01	0.98 (0.84-1.15)	8.33E-01	1.11 (0.96-1.29)	1.51 E-01	0.26	0.24	1.85E-01
rs17609940	CHD	ANKS1A	G	1.07 (1.05-1.10)	1.00 E-08	C/G	1.03 (0.9-1.17)	7.02 E-01	0.96 (0.81-1.13)	6.09E-01	1.08 (0.93-1.26)	3.16 E-01	0.20	0.21	3.52E-01
rs1122608	CHD	LDLR	G	1.14 (1.09-1.18)	1.00 E-09	T/G	0.88 (0.77-1.01)	7.32 E-02	0.93 (0.78-1.11)	4.11E-01	0.83 (0.71-0.98)	2.49 E-02	0.20	0.19	3.98E-01
rs12526453	CHD	PHACTR1	C	1.1 (1.06-1.13)	1.00 E-09	G/C	0.9 (0.8-1.01)	8.29 E-02	0.89 (0.76-1.04)	1.58E-01	0.9 (0.78-1.04)	1.60 E-01	0.27	0.27	9.66E-01
rs216172	CHD	SMG6, SRR	C	1.07 (1.05-1.09)	1.00 E-09	C/G	1.02 (0.91-1.14)	7.62 E-01	0.95 (0.82-1.1)	4.69E-01	1.06 (0.93-1.21)	4.08 E-01	0.33	0.37	5.09E-02

SNP	Trait	Reported				Effect allele/Other allele	MI		STEMI		NSTEMI		Effect allele frequency		
		Gene(s)	Effect allele	OR (95% CI)	P[a]		OR (95% CI)	P	OR (95% CI)	P	OR (95% CI)	P	STEMI	NSTEMI	X^2 P[b]
rs46522	CHD	UBE2Z, GIP, ATP5G1, SNF8	T	1.06 (1.04-1.08)	2.00E-08	T/C	1.05 (0.94-1.17)	3.56E-01	1.05 (0.92-1.21)	4.59E-01	1.05 (0.92-1.19)	4.89E-01	0.45	0.44	7.14E-01
rs599839	CHD	SORT1	A	1.11 (1.08-1.15)	3.00E-10	A/G	1.04 (0.91-1.19)	5.25E-01	0.98 (0.82-1.16)	7.89E-01	1.1 (0.94-1.28)	2.45E-01	0.21	0.20	4.79E-01
rs3798220	CHD	LPA	C	1.51 (1.33-1.70)	3.00E-11	C/T	1.06 (0.61-1.82)	8.46E-01	1.09 (0.54-2.24)	8.04E-01	1.07 (0.56-2.05)	8.41E-01	0.01	0.01	9.48E-01
rs12936587	CHD	RASD1, SMCR3, PEMT	G	1.07 (1.05-1.09)	4.00E-10	A/G	0.95 (0.85-1.07)	4.25E-01	0.93 (0.81-1.08)	3.51E-01	0.97 (0.85-1.11)	6.94E-01	0.34	0.35	6.50E-01
rs9982601	CHD	MRPS6	T	1.18 (1.12-1.24)	4.00E-10	T/C	1.11 (0.95-1.3)	1.83E-01	1.16 (0.95-1.42)	1.45E-01	1.09 (0.91-1.32)	3.46E-01	0.15	0.15	8.36E-01
rs579459	CHD	ABO	C	1.1 (1.07-1.13)	4.00E-14	C/T	1.22 (1.07-1.39)	2.47E-03	1.31 (1.11-1.56)	1.56E-03	1.15 (0.99-1.34)	7.28E-02	0.25	0.24	3.29E-01
rs17114036	CHD	PPAP2B	A	1.17 (1.13-1.22)	4.00E-19	G/A	0.82 (0.69-0.98)	3.01E-02	0.97 (0.78-1.21)	8.11E-01	0.74 (0.6-0.92)	6.05E-03	0.11	0.09	1.97E-02
rs4773144	CHD	COL4A1, COL4A2	G	1.07 (1.05-1.09)	4.00E-09	G/A	1.08 (0.96-1.21)	2.16E-01	1.1 (0.95-1.28)	1.92E-01	1.03 (0.9-1.18)	7.01E-01	0.44	0.43	5.47E-01
rs1231206	CHD	Intergenic	A	1.07 (1.05-1.09)	9.00E-10	A/G	1.02 (0.91-1.14)	7.49E-01	0.95 (0.82-1.1)	4.76E-01	1.06 (0.93-1.21)	4.00E-01	0.33	0.37	5.13E-02
rs11556924	CHD	ZC3HC1	C	1.09 (1.07-1.12)	9.00E-18	T/C	0.89 (0.78-1.01)	7.74E-02	0.85 (0.72-1.01)	6.08E-02	0.92 (0.79-1.08)	3.13E-01	0.27	0.29	1.64E-01
rs1412444	CHD	LIPA	T	1.1 (1.07-1.14)	4.00E-08	T/C	1.12 (1.01-1.25)	3.74E-02	1.09 (0.95-1.26)	2.20E-01	1.15 (1.01-1.31)	4.14E-02	0.42	0.42	8.80E-01
rs7865618	CHD	MTAP	A	1.18 (1.14-1.21)	2.00E-27	A/G	1.13 (1.01-1.26)	3.24E-02	1.13 (0.98-1.3)	9.60E-02	1.14 (1-1.29)	4.71E-02	0.40	0.39	7.37E-01
rs1333049	CHD	Intergenic	C	1.27 (1.23-1.31)	7.00E-58	C/G	1.27 (1.14-1.43)	2.87E-05	1.24 (1.08-1.44)	3.06E-03	1.29 (1.13-1.48)	2.17E-04	0.45	0.45	9.56E-01
rs9349379	CHD	PHACTR1	?	NR	9.00E-26	G/A	1.22 (1.1-1.36)	2.57E-04	1.2 (1.04-1.38)	1.26E-02	1.23 (1.09-1.4)	1.26E-03	0.47	0.48	8.88E-01
rs944797	CHD	CDKN2A, CDKN2B	C	1.25 (1.18-1.31)	6.00E-16	C/T	1.29 (1.15-1.43)	6.31E-06	1.28 (1.11-1.47)	5.49E-04	1.28 (1.13-1.46)	1.70E-04	0.48	0.47	6.89E-01

SNP	Trait	Reported				Effect allele/Other allele	MI		STEMI		NSTEMI		Effect allele frequency		
		Gene(s)	Effect allele	OR (95% CI)	P[a]		OR (95% CI)	P	OR (95% CI)	P	OR (95% CI)	P	STEMI	NSTEMI	X^2 P[b]
rs671	CHD	ALDH2	A	1.43 (1.35-1.51)	2.00 E-34	-/-	-	-	-	-	-	-	-	-	-
rs3869109	CHD	HCG27, HLA-C	G	1.14 (NR)	1.00 E-09	G/A	1.04 (0.91-1.18)	5.74 E-01	1.02 (0.87-1.21)	7.71E-01	1.07 (0.92-1.25)	3.51 E-01	0.35	0.34	5.59E-01
rs6905288	CHD	VEGFA	T	1.23 (NR)	7.00 E-08	A/G	1.11 (0.99-1.25)	6.50 E-02	1.05 (0.91-1.22)	5.07E-01	1.17 (1.02-1.34)	2.14 E-02	0.44	0.42	1.12E-01
rs9349379	CHD	PHACTR1	G	1.187 (1.22-1.44)	6.00 E-12	G/A	1.22 (1.1-1.36)	2.57 E-04	1.2 (1.04-1.38)	1.26E-02	1.23 (1.09-1.4)	1.26 E-03	0.47	0.48	8.88E-01
rs9349379	CHD	PHACTR1	G	1.338 (1.22-1.47)	8.00 E-10	G/A	1.22 (1.1-1.36)	2.57 E-04	1.2 (1.04-1.38)	1.26E-02	1.23 (1.09-1.4)	1.26 E-03	0.47	0.48	8.88E-01
rs1842896	CHD	GUCY1A3	T	1.14 (1.10-1.19)	1.00 E-11	T/G	1.1 (0.99-1.23)	8.40 E-02	1.06 (0.92-1.22)	4.47E-01	1.13 (0.99-1.28)	7.42 E-02	0.43	0.41	3.60E-01
rs9349379	CHD	PHACTR1	G	1.15 (1.10-1.21)	2.00 E-09	G/A	1.22 (1.1-1.36)	2.57 E-04	1.2 (1.04-1.38)	1.26E-02	1.23 (1.09-1.4)	1.26 E-03	0.47	0.48	8.88E-01
rs7136259	CHD	ATP2B1	T	1.11 (1.08-1.15)	6.00 E-10	C/T	0.97 (0.86-1.09)	6.06 E-01	0.96 (0.82-1.12)	6.10E-01	0.99 (0.86-1.14)	8.98 E-01	0.30	0.30	8.49E-01
rs2123536	CHD	TTC32, WDR35	T	1.12 (1.08-1.16)	7.00 E-11	C/T	0.96 (0.78-1.19)	7.31 E-01	1.15 (0.87-1.52)	3.20E-01	0.9 (0.71-1.14)	3.84 E-01	0.07	0.09	2.32E-02
rs10757274	CHD	CDKN2A/B	G	1.37 (1.31-1.43)	8.00 E-45	G/A	1.28 (1.15-1.43)	7.18 E-06	1.25 (1.09-1.44)	1.55E-03	1.3 (1.14-1.48)	6.15 E-05	0.48	0.47	9.30E-01
rs11066280	CHD	C12orf51	A	1.19 (1.13-1.25)	2.00 E-11	-/-	-	-	-	-	-	-	-	-	-
rs9268402	CHD	C6orf10, BTNL2	G	1.16 (1.12-1.20)	3.00 E-15	-/-	-	-	-	-	-	-	-	-	-

[a] Originally reported association statistics

[b] X^2 test P-value for the allele frequency difference between NSTEMI and STEMI cases

MI, myocardial infarction; CHD, coronary heart disease; EA, effect allele; OA, other allele; EAF, effect allele frequency; STEMI, ST elevation MI; NSTEMI non-ST elevation MI

Supplementary Table B. Genotyping arrays for the FINRISK participants of the prospective replication sample II by sampling year and disease endpoint. All numbers reported before genotype quality control and sample exclusions.

Genotyping Array	Year				Incident MI		Incident NSTEMI		Incident STEMI	
	2002	2007	1992	1997	unaffected	affected	unaffected	affected	unaffected	affected
Affymetrix Genome-Wide Human SNP Array 6.0	52	0	58	56	149	17	164	2	162	4
Illumina Human610-Quad	616	719	0	546	1844	37	1865	16	1877	4
Illumina HumanCoreExome-24	3415	3473	3518	0	10313	93	10371	35	10387	19
Illumina HumanOmniExpress 12v1	1396	84	646	4864	6575	415	6862	128	6903	87

MI, myocardial infarction; STEMI, ST elevation myocardial infarction; NSTEMI, non-ST elevation myocardial infarction
